# Evaluation of Process Parameters for Integrated CO_2_ Electrolysis to Produce Ethylene

**DOI:** 10.1002/open.202500611

**Published:** 2026-02-01

**Authors:** Fabian Hauf, Ricarda Kollmuß, Stefan Haufe, Elias Klemm

**Affiliations:** ^1^ Institute of Technical Chemistry University of Stuttgart Stuttgart Germany; ^2^ Consortium für Elektrochemische Industrie Wacker Chemie AG München Germany

**Keywords:** bicarbonat electrolyzer, C–C coupling, integrated CO_2_ electrolysis, process parameters

## Abstract

Electrochemical CO_2_ reduction provides a promising strategy for reducing greenhouse gas emissions by converting CO_2_ into chemicals such as ethylene. Integrated CO_2_ electrolysis, using CO_2_‐enriched absorbent solutions, is a cost‐effective alternative to gas‐fed systems due to reduced process complexity. However, for industrial applications, the process parameters need to be optimized to enhance selectivity and efficiency. Despite advances in catalyst and cell design, the impact of operational factors like catholyte flow rate, pressure, and temperature on C_2+_ product selectivity remains largely unexplored. This study systematically investigates the effects of catholyte flow rate, overpressure, and temperature on ethylene selectivity in integrated CO_2_ electrolysis with a potassium carbonate absorbent. Our results show that increasing the catholyte flow rate enhances the Faraday efficiency for ethylene by mitigating mass transport limitations between the flow field and the catalyst layer, whereas increasing pressure or temperature does not yield similar improvements. This insight shifts the focus from stoichiometric availability of physically dissolved CO_2_ to mass transport limitations, suggesting that further advances in cell design could unlock higher conversion efficiencies. Our study provides a foundation for scaling up integrated CO_2_ electrolysis by highlighting the importance of improving mass transport, a key step toward industrial implementation of sustainable CO_2_ conversion technologies.

## Graphical abstract

This work systematically explores how flow rate, pressure, and temperature influence ethylene selectivity in integrated CO_2_ electrolysis with a potassium carbonate absorbent. Findings reveal that higher catholyte flow rates improves Faraday efficiency for ethylene by overcoming mass transport barriers, while increased pressure or temperature does not. This highlights mass transport as a key factor, informing strategies for scaling up integrated CO_2_ electrolysis and advancing sustainable CO_2_ utilization.

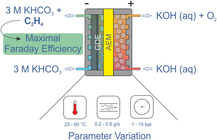

## 1 Introduction

With the ongoing progression of global warming, the need for innovative strategies to reduce greenhouse gas emissions has become increasingly urgent. A critical aspect of this challenge is the mitigation of hard‐to‐abate emissions, such as those originating from waste incineration plants and cement production [1]. Electrochemical CO_2_ reduction has emerged as a promising technology to address this issue by enabling the direct conversion of CO_2_ into valuable chemicals and fuels. By closing the carbon cycle, this approach not only reduces CO_2_ emissions but also supports the development of a circular economy through the production of platform chemicals. Among these, ethylene (C_2_H_4_) is particularly attractive due to its widespread industrial use and substantial global market demand [2, 3].

Currently, various setups for CO_2_ electroreduction are under investigation, which can be generally categorized based on the form in which CO_2_ is supplied to the electrochemical cell. Specifically, the CO_2_ feed can be introduced either in a gaseous state or as a dissolved species. In the gaseous approach, CO_2_ is first captured from flue gas, then desorbed from the capture medium, and subsequently fed as a gas into the electrolyzer [4, 5, 6]. To avoid the cost‐intensive desorption step, an alternative method known as integrated CO_2_ electrolysis has been developed. In this approach, the CO_2_‐enriched absorbent solution is directly introduced into an electrolyzer without prior desorption [7, 8]. This integrated strategy is expected to simplify the overall process and reduce production costs [9, 10].

Recent advancements in integrated CO_2_ electrolysis have been reported with Faraday efficiencies (FE) to C_2_H_4_ of more than 20% [7, 8]. While previous studies have primarily focused on catalyst development and cell design [8, 11, 12, 13], the influence of electrolysis process parameters is equally critical. These parameters can substantially affect various performance indicators and reaction conditions within the electrolyzer cell, including the supply of reactants to the catalyst, thereby mitigating mass transport limitations, as well as ensuring an efficient removal of products from the catalyst surface.

One of the key parameters that can be adjusted in integrated CO_2_ electrolysis is the absorbent solution. It is essential that the CO_2_‐loaded absorbent solution provides a sufficient supply of electrochemically accessible CO_2_ to the catalyst to achieve high conversion rates. Simultaneously, the absorbent must be compatible with the electrolysis requirements, including membrane compatibility, stability within the electrochemical window, and adequate conductivity to ensure efficient operation. We focus in this study on a CO_2_‐saturated potassium bicarbonate solution, previously identified alongside amine‐based absorbents as promising candidates for integrated CO_2_ electrolysis [14].

For C_1_ products, numerous studies have investigated the optimization of operational parameters, such as temperature, pressure, and the flow rate of CO_2_‐saturated catholyte, to maximize product yields. Increasing the temperature, for example, has been shown to enhance CO production in integrated CO_2_ electrolysis systems [15, 16]. This improvement is attributed to a combination of factors, including accelerated reaction kinetics and increased CO_2_ liberation within the electrolyzer cell. Similarly, higher temperatures have been reported to increase the FE for formic acid production [17].

The flow rate of the electrolyte through the cathodic compartment also plays a crucial role, as it directly affects both the delivery of reactants to, and the removal of products from, the catalyst surface. This, in turn, shapes the local environment at the catalyst. For instance, Xiao et al. demonstrated that the impact of flow rate on CO production is dependent on the applied current density [18], while Nomoto et al. observed that the FE for formate increases with higher flow rates [19].

Achieving industrially relevant current densities is essential for the practical conversion of CO_2_. Larrea et al. reported that, for CO production, increasing the current density leads to higher single‐pass conversion but results in a decrease in CO FE, likely due to CO_2_ depletion and the subsequent dominance of the hydrogen evolution reaction (HER). A similar trend of decreasing FE with increasing current density has been observed for formate [19].

To address the formation of less C_1_ products at higher current densities, increasing the system pressure has proven to be an effective approach. For example, Zishuai et al. demonstrated that elevating the pressure from 1 to 4 atm increased the CO FE from 55% to 95%, which is attributed to an increased supply of CO_2_ to the catalyst [15]. Thus, increasing the pressure offers a means to counteract FE losses during CO_2_ electrolysis.

These parameter studies highlight that process conditions can have a significant impact on the performance indicators for C_1_ product formation. However, to date, the influence of such process parameters on the formation of C_2+_ products in integrated CO_2_ electrolysis systems has not been intensively studied. Current studies often focus on varying the current density, with higher current densities reported to decrease C_2+_ product formation [7, 8]. This decrease can likely be attributed to mass transport limitations and thus a reactant depletion at the catalyst layer. Studies beyond the current density are needed to understand and optimize additional process parameters such as temperature, pressure, and flow rate, as their effects on C_2+_ product yields remain unclear. Optimizations in these areas are essential for enhancing C_2_H_4_ production on a larger scale and achieving the industrial viability of CO_2_ electrolysis.

This work systematically evaluates how mass transport limitations can be mitigated by optimizing process parameters, specifically catholyte flow rate, overpressure, and temperature, in integrated CO_2_ electrolysis to C_2_H_4_ from saturated KHCO_3_ electrolyte. By investigating the relationship between these operating conditions and product selectivity, we provide detailed insights into the impact of process optimization on system performance. The data indicate that increased convective mass transport is particularly effective in boosting the carbon conversion rate.

## 2 Results and Discussion

**FIGURE 1:**
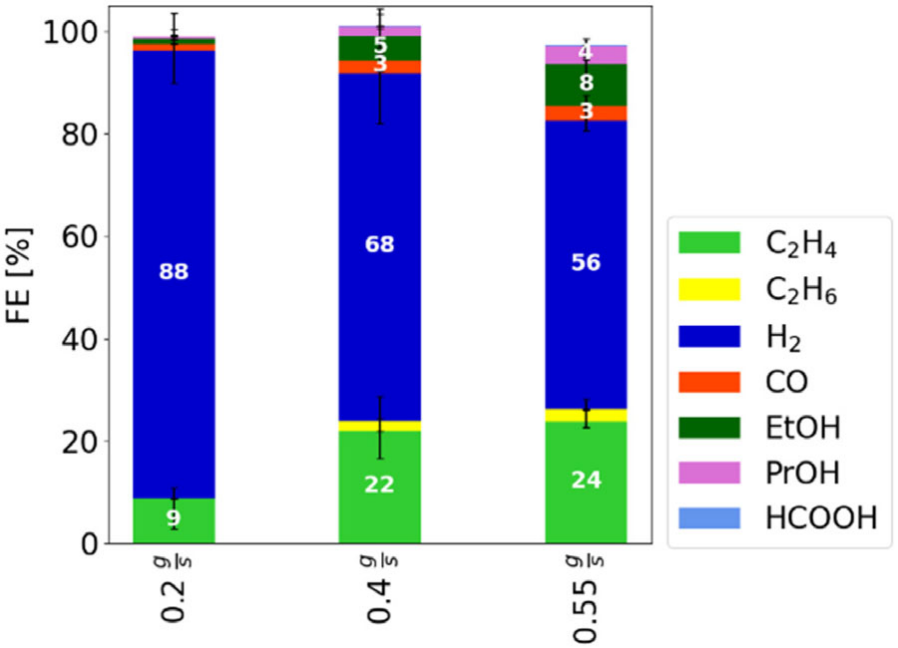
Variation of the catholyte flow rate through the electrolyzer cell in the range of 0.2–0.55 g s^−1^, whereby the FE for carbon‐containing products increases with higher flow rates. Each bar represents the median of the FE of three independent experiments after 3.5 h of electrolysis with the range indicated by the error bars. The electrolysis was performed at 1 bar(a) (absolute pressure), 25°C, a current density of 50 mA cm^−2^, and in the standard electrolyzer setup.

According to previous studies, mass transport limitations are evident not only at elevated current densities but also at lower current densities, such as 50 mA cm^−2^, where the HER can already be dominant in integrated CO_2_ electrolysis [14]. To address these limitations, a logical approach is to vary the catholyte flow rate, thereby adjusting the supply of CO_2_ to the cell. Figure 1 shows the FE of CO_2_ electrolysis at different catholyte pump flow rates, specifically examining the range from 0.2 to 0.55 g s^−1^. Higher flow rates were not considered, as they would be challenging to implement in a scaled‐up electrolyzer system without having an intolerable pressure drop in the system [20].

The experiments show that H_2_ remains the dominant product at all three flow rates. Nevertheless, increasing the flow rate reduces the HER by 36%, with a corresponding increase in the formation of carbon‐containing products. These products include C_2_H_4_, C_2_H_6_, CO, EtOH, PrOH, and HCOOH, with C_2_H_4_ accounting for more than 50% of the FE for carbon‐containing products. The highest yields for these products were observed at the maximum tested flow rate of 0.55 g s^−1^.

Although an increase in C_2_H_4_ FE was observed, residual CO_2_ remained in the off‐gas across all experiments. This indicates incomplete CO_2_ conversion during electrolysis. Potential causes include mass transport issues or catalyst deactivation. Previous studies have shown that the same electrolysis cell, when operated in a gaseous mode, achieves higher efficiencies for carbon‐containing products [21], suggesting that the cell is capable of higher conversions with adequate CO_2_ supply. Rottmann et al. further demonstrated superior CO_2_ conversion efficiencies for the same catalyst in a gaseous configuration [22]. Thus, catalyst deactivation or intrinsic cell limitations are unlikely, reinforcing the hypothesis that CO_2_ supply is the limiting factor. These limitations can arise from CO_2_ depletion in different regions of the cell. If all electrochemically available CO_2_ had reacted within the cell, a depletion of CO_2_ in the electrolyte toward the outlet of the catholyte flow field would be expected, representing a stochiometric limitation. Here, a concentration gradient in the electrolyte between the inlet and the outlet of the cell is created.

Alternatively, when the absorbent solution leaving the cell contains a sufficient amount of physically dissolved CO_2_ that cannot be electrochemically converted, the available CO_2_ will simply pass through the cell without participating in the reaction. This situation exemplifies mass transport limitation, which is marked by concentration gradients between the catalyst layer and the flow field. The mass transport process along this gradient can be spatially divided into three main regions: the macropores layer, the microporous layer, and the catalyst layer (see Figure S1 in Supporting Information). It can be assumed that gas and liquid coexist in all these layers.

To gain detailed insights into the kinetics of mass transport in these layers, operando 3D imaging techniques would have been necessary, which are very challenging. However, such measurements are not feasible in this study due to the use of a commercial zero‐gap cell (see Section 4 “Experimental Section”). Nonetheless, the overall electrochemical cell performance can be interpreted in terms of the three main driving forces outlined in the Nernst–Planck equation [23]: diffusion, migration, and convection. Among these driving forces, migration is of minor importance in this context, as physically dissolved CO_2_ is an uncharged molecule. Convection plays a central role in the present electrochemical cell, given that the feed is introduced perpendicular to the flow field (see Figure S2 in Supporting Information). This configuration suggests that the absorbent solution is transported into the macropore layer of the gas diffusion electrode (GDE) via convection at the inlet. In contrast, diffusion becomes increasingly significant within the microporous layer, where the denser structure restricts convective transport. The gas produced at the catalyst layer subsequently moves in a counterflow direction, traveling from the catalyst layer back toward the flow field. However, if local gas evolution is too strong, gas can also escape through seals and the anode side.

Varying the catholyte flow rate can mitigate both stochiometric limitation in the flow field and mass transport limitation in the microporous layer. An increased flow rate delivers more CO_2_ to the system per unit time, thereby alleviating stochiometric limitations by ensuring a sufficient supply of reactant throughout the whole catholyte flow field. Moreover, higher flow rates facilitate convection by increasing the depth of penetration of the incoming stream into the microporous layer as well as the velocity in the flow field. This leads to reduced concentration gradients and, consequently, lowers mass transport limitations. Given that CO_2_ constitutes more than 60% of the off‐gas (excluding the carrier gas), it is reasonable to assume that the absorbent contains sufficient physically absorbed CO_2_. Moreover, the lambda factor, defined as the ratio of the physically absorbed CO_2_ supplied by the absorbent to the CO_2_ required for conversion to C_2_H_4_, remains sufficient to provide an excess of CO_2_ supply even at the lowest flow rate (see Table 1). Therefore, a stoichiometric limitation is unlikely to be the primary constraint on carbon‐containing product formation. Rather, enhanced mass transport, resulting from deeper penetration into the macropore layer and elevated flow velocities, appears to promote the conversion to carbon‐containing products. Additionally, the nonlinear pressure drop observed (see Table 1) lends further support to the hypothesis that a greater penetration depth is observed with higher flow rates, as this would lead to an increased transport path within the macropores layer. Unlike findings reported elsewhere [24], the pressure drop at the cell entrance in this study does not lead to CO_2_ degassing into the gas diffusion layer (GDL), as the absorbent solution is saturated at system pressure (1 bar(a)) and the pump increases the pressure at the inlet of the cell according to the pressure drop needed to flow through the cell. These observations collectively indicate that mass transport limitations are the dominating factor affecting the FEs of carbon‐containing products.

**TABLE 1:** Overview of the pressure drop between the catholyte inlet (generated by the pump) and the outlet (system pressure), as well as the lambda factor for the CO_2_ concentration of the bulk electrolyte to C_2_H_4_, during electrolysis experiments conducted at various flow rates (compare Figure 1). The pressure drops reported represent the time‐averaged pressure drops over the entire duration of each experiment. The ranges provided in brackets indicate the variability of the time‐averaged pressure drops observed in each individual experiment.

Catholyte flow rate, g s^−1^	0.2	0.4	0.55
Pressure drop (mbar)	210 [200 to 230]	480 [450 to 510]	700 [680 to 720]
Lambda factor for C_2_H_4_	4	10	14

To further examine the hypothesis that mass transport limitations are predominant in our electrolysis cell, additional experiments were performed under elevated pressure conditions. Similar to the effect of increasing the flow rate, raising the pressure enhances the amount of physically dissolved CO_2_ in the electrolyte, thereby allowing more accessible CO_2_ to be supplied from the absorbent to the cell [25]. This approach is expected to particularly mitigate stoichiometric limitations. Moreover, the increased CO_2_ concentration influences mass transport, as both convection and diffusion are related to the concentration of CO_2_.

**FIGURE 2:**
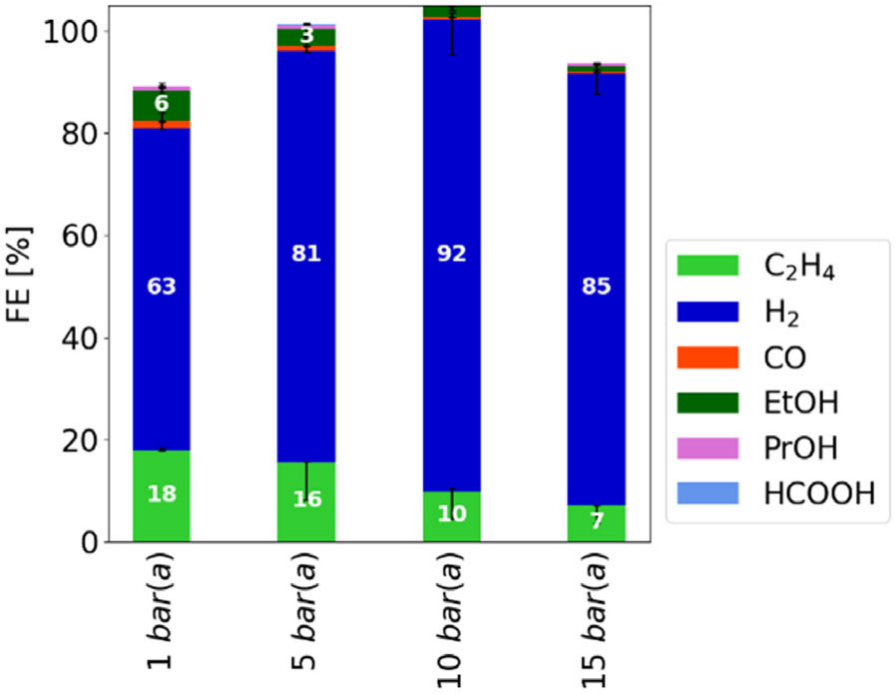
Variation of the overpressure during integrated CO_2_ electrolysis between 1 and 15 bar(a), whereby the FE shows a decreasing trend to carbon containing products with increasing pressure. Each bar represents the median of the FE of three independent experiments after 3.5 h of electrolysis with the range indicated by the error bars. The electrolysis was performed at 25°C, a catholyte flow rate of 0.4 g s^−1^, a current density of 100 mA cm^−2^ and in the standard electrolyzer setup.

Figure 2 presents the FE of CO_2_ electrolysis at overpressures ranging from 1 to 15 bar(a) (absolute pressure). This pressure range was selected because it remains within the linear regime of the concentration of physically absorbed CO_2_ within the absorbent solution [26], allowing for identification of clear correlations. According to Henry's law, the CO_2_ load of the absorbent solution should increase in this range from 21 to 321 mmol L^−1^ [14].

The product distribution observed in these measurements was consistent with previous experiments, with H_2_ remaining the predominant product, with FEs greater than 60%. The FE of carbon‐containing products, mainly CO, C_2_H_4_, and EtOH, exhibited a decreasing trend with increasing pressure. In particular, C_2_H_4_ decreases from an FE of 18% to 7%.

Our results demonstrate that the increased concentration of available CO_2_ in the absorbent solution does not necessarily exhibit a positive correlation with the FE for carbon‐containing products. This indicates that, under the current electrolysis conditions, the total accessible CO_2_ within the cell is not the limiting factor. This conclusion is further supported by the consistent detection of significant amounts of CO_2_ in the off‐gas across all experiments, suggesting that CO_2_ conversion is not substantially constrained by stoichiometric limitations.

Increasing the pressure raises the CO_2_ concentration in the absorbent; however, it also reduces the volume of product gas. This reduction is due to both increased gas solubility in the absorbent solution and the compression of the gas at constant molar quantity. As a result, fewer or smaller gas bubbles are formed. Since the product gas must traverse the catalyst layer and the microporous layer before reaching the flow channel, the lower gas volume diminishes the mixing that occurs during this passage. As a result, the reduction in bubble‐induced mixing may hamper mass transport and ultimately decrease CO_2_ conversion.

**FIGURE 3:**
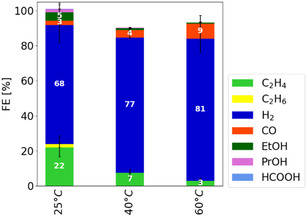
Variation of the temperature during integrated CO_2_ electrolysis between 25°C and 60°C. The FE show a decreasing trend to carbon containing products with increasing temperature. Each bar represents the median of the FE of three independent experiments after 3.5 h of electrolysis with the range indicated by the error bars. The electrolysis was performed at 1 bar(a), a catholyte flow rate of 0.4 g s^−1^, a current density of 50 mA cm^−2^ and in the standard electrolyzer setup.

To increase the amount of accessible CO_2_ at the catalyst layer, raising the temperature of the absorbent solution could promote the diffusion of physically absorbed CO_2_, thereby improving mass transport. Higher diffusivity enables CO_2_ from deeper within the flow field channel to reach the catalyst layer, allowing more CO_2_ from the enriched absorbent solution to access the electrochemically active sites and undergo reduction. However, this effect is counteracted by the reduced solubility of CO_2_ in the absorbent solution at higher temperatures. Figure 3 systematically examines these temperature effects by presenting the FE of CO_2_ electrolysis at temperatures ranging from 25°C to 60°C. This temperature range was chosen because in a scale‐up of the system to the stack level, cooling below room temperature is impractical, while temperatures up to 60°C are considered optimal for heat utilization in electrolyzers [27].

The data show that with increasing temperature, the FE for H_2_ increases, reaching 81% at 60°C. Consequently, the expected increase in carbon‐containing product formation is not achieved. Furthermore, the FE for C_2+_ products, mainly C_2_H_4_, C_2_H_6_, EtOH, and PrOH, decreases, with C_2_H_4_ dropping from 22% to 3%. Although elevated temperatures should increase the diffusivity of CO_2_, a reverse effect was observed. This effect can be attributed to the decrease in the CO_2_ solubility in the absorbent solution, which influences the concentration gradient across the microporous layer. Nevertheless, a depletion of CO_2_ already in the electrolyte and thus a stochiometric limitation is not observed in this temperature range, as residual CO_2_ is still detectable in the off‐gas.

While the FE for C_2+_ products decreases, the FE for CO increases from 3% to 9% as the temperature rises. This suggests that at elevated temperatures, the dimerization of CO is less favored, producing less C_2+_ products. In the literature, the influence of temperature on CO formation during CO_2_ electrolysis to C_1_ products has been reported to result in an increase in the CO FE of ≈25% when the temperature is raised from 20°C to 60°C [15]. In contrast, the observed increase in the CO FE of 300% in this study is significantly higher.

This discrepancy may be attributed to the broader product distribution associated with the copper catalyst, which, at lower temperatures, results in a CO FE of 3% on copper compared to 59% on silver [15]. Since CO is a known intermediate for higher‐order carbon products, dimerization on copper reduces the total CO FE in favor of C_2+_ formation. Consequently, the relative increase appears to be quite different, primarily driven by the low CO FE observed at lower temperatures.

In addition to changes in FE, a decrease in cell voltage was observed with increasing temperature, as shown in Table 2. This reduction in cell voltage can be attributed to several factors, including enhanced electrochemical reaction kinetics, decreased membrane resistivity, and improved electrolyte conductivity. The effect of temperature on reaction kinetics can be evaluated using impedance spectroscopy. However, since the experimental setup employs a zero‐gap flow cell with predominantly metallic components, impedance measurements do not provide meaningful information regarding electrolyte resistance. Besides reaction kinetics, the observed decrease in cell voltage may be due to several factors, such as the enhanced mobility of ionic species at elevated temperatures. Additional studies are necessary to determine the specific impact of each participating component on the total cell potential.

**TABLE 2:** Summary of the cell voltage, anolyte, and catholyte conductivity at the different temperatures tested for the experiments shown in Figure 3. The reported cell voltages are the average values in time between 3 and 3.5 h of electrolysis time.

Electrolyte Temperature, °C	25	40	60
Cell Voltage (V)	5.9 (5 to 6.4)	5.5 (5.3 to 5.7)	4.2 (4.1 to 4.3)

Of the three investigated process parameters, flow rate, overpressure, and temperature, only the flow rate of the catholyte was found to have a positive impact on the limiting mass transport phenomena during the integrated CO_2_ electrolysis at 50 mA cm^−2^. Consequently, improvements in the FE for carbon‐containing products, particularly C_2_H_4_, were observed only under increased flow rate conditions compared to the original conditions. For scaling up the process, it is vital to increase the current density in order to reduce the electrolyzer size and thus investment costs while maintaining CO_2_ conversion rates. To investigate whether increasing the flow rate remains effective for boosting carbon product yields at higher current densities, additional experiments with different flow rates were carried out. The current density was raised to 100 mA cm^−2^, as at a higher level, the additional gas evolution (predominantly hydrogen) may not be confined to the cathode outlet, making leakage currents a relevant factor (for example, through the membrane). Details of the current density variation can be found in Figure S3 in the Supporting Information.

**FIGURE 4:**
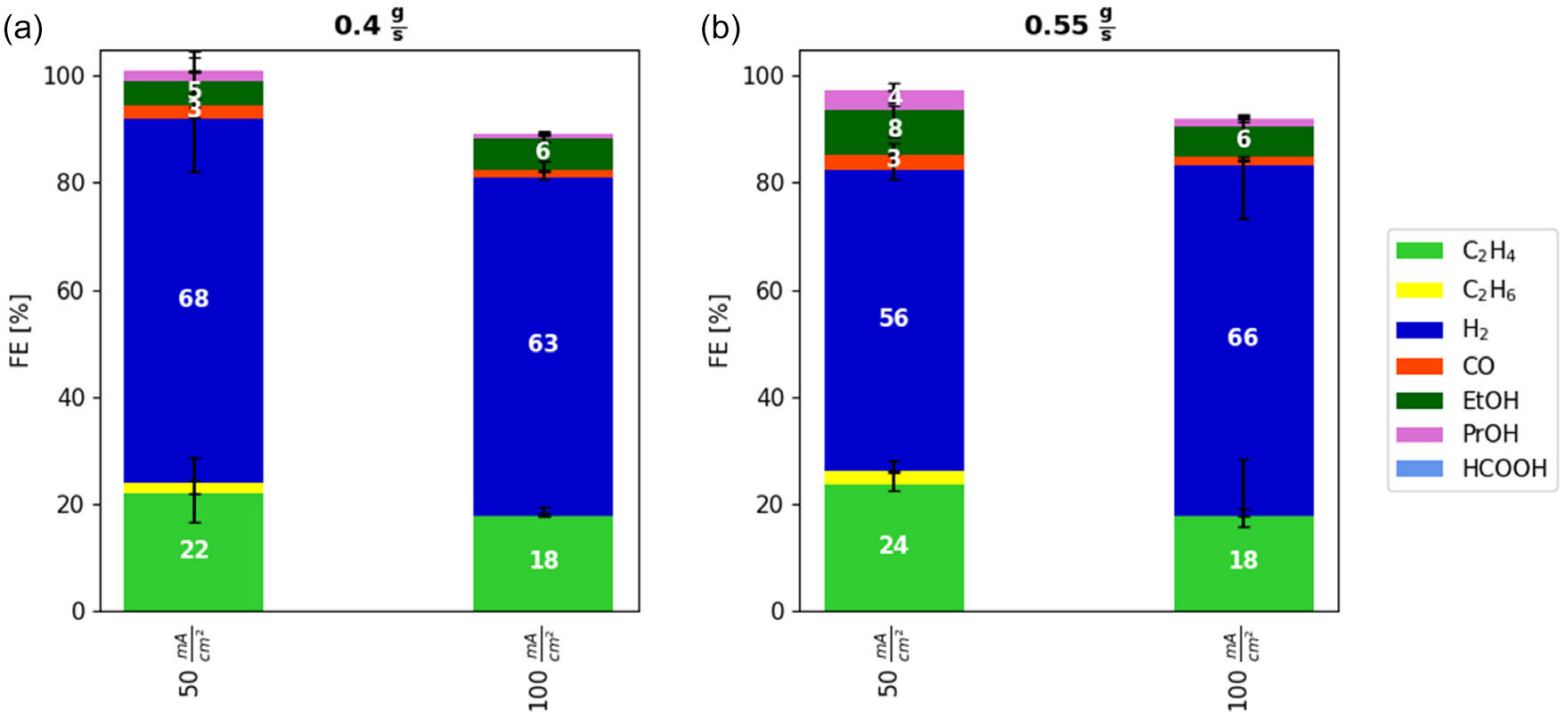
Variation of the current density from 50 to 100 mA cm^−2^ at catholyte flow rates of (a) 0.4 g s^−1^ and (b) 0.55 g s^−1^. The data show that the increase in the current density decreases the FE for carbon‐containing products. Each bar represents the median of the FE of three independent experiments after 3.5 h of electrolysis with the range indicated by the error bars. The electrolysis was performed at 1 bar(a), 25°C, and the in standard electrolyzer setup.

Figure 4a,b illustrate that, for both tested flow rates (0.4 and 0.55 g s^−1^), an increase in current density from 50 to 100 mA cm^−2^ causes a decline in the FE of carbon‐containing products. However, the anticipated halving of FE under mass transport‐limited conditions, assuming similar transport rates at both current densities, is not observed. This discrepancy may be due to variations in experimental conditions, such as enhanced gas evolution limiting the CO_2_ supply or higher cell voltages occurring at increased current densities.

Furthermore, comparison of Figures 4a,b demonstrates that, at 100 mA cm^−2^, increasing the flow rate from 0.4 to 0.55 g s^−1^ does not affect the FE for C_2_H_4_, which contrasts with the trend observed at 50 mA cm^−2^. This suggests that the additional convection provided by the higher flow rate does not significantly enhance CO_2_ supply at this increased current density. The observed outcome likely arises from multiple contributing factors arising from changes in the local environment. While an increase in current density generally leads to a higher working potential, this change is not thought to significantly affect the insensitivity to flow rate. In contrast, the greater gas evolution at elevated current densities intensifies bubble‐induced mixing in the macroporous layer, which may become the dominant effect compared to changes in flow rate. Although this mechanism is expected to be predominant, the influence of other factors cannot be entirely ruled out.

The optimal CO_2_ reduction FEs at 50 mA cm^−2^ in this work are in line with previous studies, with C_2_H_4_ FEs at 100 to 200 mA cm^−2^ reported between 20% and 30% [7, 8]. At 100 mA cm^−2^, a stronger dominance of the HER is observed here, suggesting that mass transport limitations are more pronounced in the present system. This variation is likely due to differences in flow field geometry, which may have alleviated such limitations in prior work.

Further investigations are needed to determine the key factors contributing to this limitation, with the objective of proposing more effective approaches to improve mass transport and achieve higher current densities. This may involve optimizing the macroporous layer to create sufficient pathways for CO_2_ delivery to the catalyst, also at conditions with increased product gas formation. Moreover, adjusting the flow field geometry, for example by employing an interdigitated configuration, could help sustain high convective mass transport and guarantee adequate CO_2_ delivery to the catalyst.

## 3 Conclusion

This empirical study provides a systematic variation of key process parameters in integrated CO_2_ electrolysis to C_2_H_4_, such as catholyte flow rate, overpressure, and temperature, and discusses strategies to address mass transport limitations. The results indicate that mass transport limitations between the channel floor of the flow field and the catalyst have a greater impact than those arising from stoichiometric limitations. Of all the parameters examined, the electrolyte flow rate exerted the greatest influence on promoting convection and mitigating mass transport constraints. Yet, at higher current densities, increasing the flow rate did not provide equivalent advantages, most likely due to the higher gas evolution shifting the mass transport in the electrochemical cell. Nevertheless, these findings clearly demonstrate that increasing the flow rate is an effective strategy for alleviating mass transport constraints, highlighting the need for future optimization of cell geometry and membrane electrode design.

In conclusion, overcoming mass transport limitations is a prerequisite for addressing stochiometric limitations. This optimization can be achieved by modifying the membrane electrode assembly or cell design to enhance mass transport within the cell. Once these improvements are realized, the trends observed for the examined parameters, such as a positive correlation with overpressure due to increased physically absorbed CO_2_, are expected to become more pronounced. Addressing these challenges will be the focus of future work.

## 4 Experimental Section

### 4.1 Materials and Chemicals

The chemicals potassium hydroxide (KOH, Carl Roth, 99.7%) and potassium bicarbonate (KHCO_3_, Carl Roth, ≥99.7%) were used as received. For all experiments, ultrapure water (18 MΩ) from an Arium mini water purification system (Sartorius) was used. Carbon dioxide (Linde, 2.5) and helium (Linde, 4.6) gases were used without any pre‐treatment.

### 4.2 Electrolysis Setup

**FIGURE 5:**
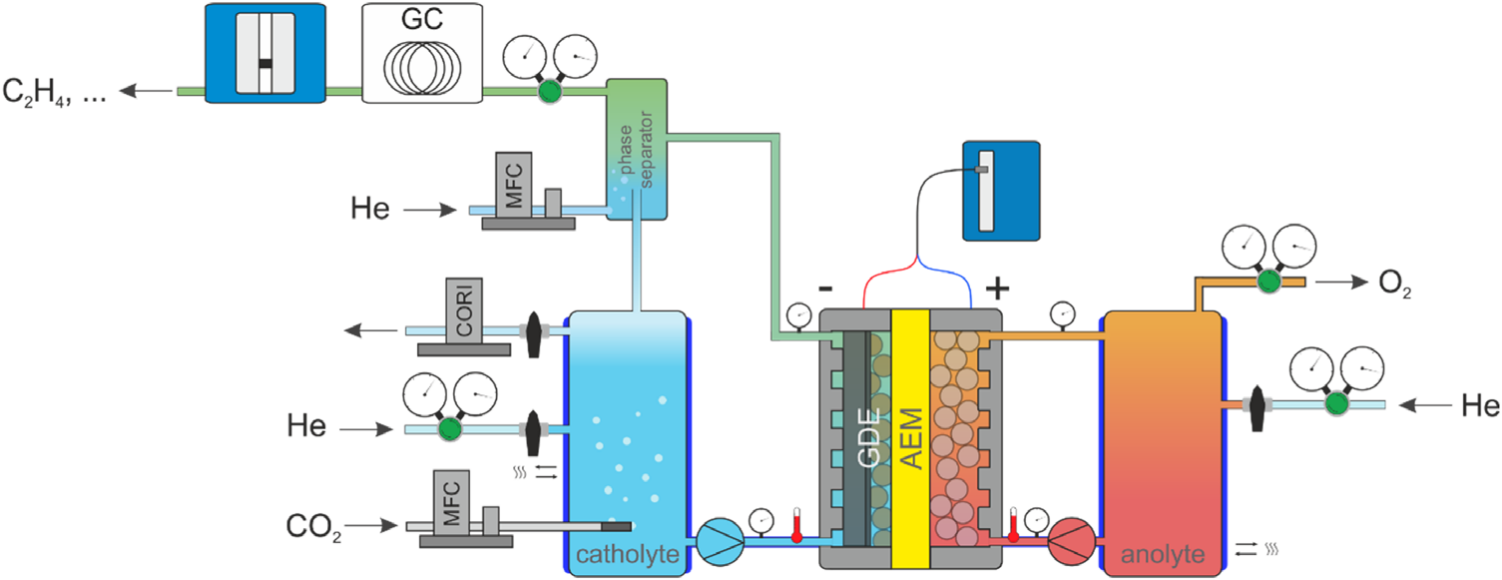
Schematic representation showing the CO_2_ electrolysis setup with a two‐compartment cell.

Parameter screening was conducted using a specially designed zero‐gap CO_2_ electrolysis setup. The system allowed for precise control of key parameters, including temperature, pressure, flow rate, and current density. Electrolysis experiments were performed in a two‐compartment cell, with liquid electrolyte circulating on both the cathodic and anodic sides. A schematic representation of the setup is shown in Figure 5.

Electrolysis was carried out in galvanostatic mode at current densities ranging from 50 to 200 mA cm^−2^, using a power supply from Elektro‐Automatik (EA‐PS 3040‐20 C). All experiments were conducted using a zero‐gap electrochemical cell supplied by eChemicles, featuring a geometric working electrode area of 8 cm^2^ [28] (see Figure S2 in Supporting Information). The cell comprised two compartments separated by an anion exchange membrane (PiperION A20, 20 µm, Versogen). The anode consisted of an iridium‐coated titanium frit provided by the cell manufacturer, while the cathode was an in‐house fabricated copper‐based GDE.

For the GDE preparation, a GDL (Freudenberg, H23 C8) was coated with copper nanoparticles (Sigma–Aldrich, 40–60 nm) and Nafion D2020 ionomer (weight ratio 8:1 Cu:Nafion) using a homemade inkjet printer (see Figure S1 in Supporting Information). A detailed description of the printing process and the GDE's preparation is given in our previous publication [14]. Each GDE with a diameter of 32 mm had a final copper and ionomer loading of ≈1 mg cm^−2^.

Temperature control was achieved by heating both electrolyte vessels using a Cryo‐compact circulator (Julabo, CF30) as well as heating the connecting pipes between the vessels and the cell, ensuring stable temperature conditions throughout the process. Each vessel contained 1 L of electrolyte, which was continuously recirculated through the cell by two micro gear pumps (HNP Mikrosystems, MoDoS). The flow rate was adjusted by varying the pump speed, allowing for a range between 0.2 and 0.55 g s^−1^, with both compartments operated at equal flow rates.

Overpressure within the system was regulated by initially pressurizing the setup with helium, as depicted in Figure 5. The pressure could be controlled within a range of 1 – 15 bar(a). During operation, two back‐pressure regulators (Swagelok) enabled independent pressure control for both the catholyte and anolyte compartments.

Multiple pressure sensors (WIKA S‐11) and temperature sensors (RS Pro Type K) positioned before and after the cell enabled precise monitoring and control of operational parameters during electrolysis. Electrical conductivity was continuously monitored by a Memosens CLS82E device from Endress+Hauser to facilitate early detection of anomalies in both electrolytes.

To ensure a sufficient supply of CO_2_ during electrolysis, the catholyte was continuously purged with CO_2_, maintaining an excess in the catholyte vessel (15–20 mLn min^−1^, controlled by a Bronkhorst FLOW‐BUS). Under ambient conditions, the excess of CO_2_ was quantified at the gas outlet of the catholyte vessel using a CORI‐FLOW mass flow meter (Bronkhorst Deutschland Nord GmbH). At elevated pressures, the excess CO_2_ was mixed with the product gas stream and directed to the gas chromatograph (GC) for analysis and quantification.

A phase separator was installed downstream of the catholyte side of the cell to facilitate the separation of gaseous products from the liquid absorbent during electrolysis. Helium was introduced into the phase separator (as depicted in Figure 5) to enhance the driving force for product gas separation, with the flow rate adjusted between 5 and 50 mLn min^−1^ (Bronkhorst FLOW‐BUS) depending on the electrolysis parameters. The product gas exited the system via a back pressure regulator and was subsequently analyzed by an in‐line GC. The volumetric flow rate was determined using a volume meter (Defender 530+ Low, MesaLabs). Prior to the start of each experiment, helium was channeled through the phase separator to maintain an air‐free atmosphere on the cathode side.

For each electrolysis experiment, 3 M KHCO_3_ was prepared as the catholyte and filled into the corresponding vessel. To ensure complete saturation, CO_2_ was bubbled through the catholyte in excess, resulting in a CO_2_‐saturated absorbent solution. The anolyte, consisting of 1 M KOH, was reused between experiments until its conductivity dropped below 100 mS cm^−1^.

CO_2_ electroreduction from the absorbent solution was performed under chronopotentiometric conditions. The cell was pre‐conditioned by ramping the current at 3.75 mA cm^−2^ min^−1^ up to the target current density. Each experiment was conducted for more than 3.5 h (including the current ramp), and every set of parameters was measured in triplicate to ensure reproducibility. Data analysis was performed using Python.

### 4.3 Product Analysis

The gaseous products generated during electrolysis were analyzed using an in‐line GC (Agilent 8860 GC System with a JAS Valve System). The GC was operated with helium as the carrier gas and was capable of detecting H_2_, CO, CO_2_, CH_4_, C_2_H_4_, C_2_H_6_, C_3_H_6_, C_3_H_8_, N_2_, and H_2_O. Higher carbon products could not be detected with the method used. Calibration was performed using two separate gas mixtures from Nippon Gases Deutschland GmbH. For further details regarding the GC analysis, please refer to our previous publication [14].

Quantification of all gaseous products was performed to determine their respective FEs. The FE was calculated using the following formula:

(1) $$FE_{i}=\frac{z_{i}\cdot c_{i}\cdot j_{n}\cdot F}{I}$$

where *FE*_*i*_ represents the FE of component *i* (e.g., C_2_H_4_), *z*_*i*_ is the number of electrons required for the formation of component *i* (*z* = 12 for C_2_H_4_), and *c*_*i*_ [vol%] is the volume fraction of the specific product in the product stream as determined by GC. *I* [A] is the current passed into the electrolyzer cell, *F* is the Faraday constant (96485 C mol^−1^), and *j*_*n*_ [mol s^−1^] is the total product stream, which can be determined by the ideal gas law as follows:

(2) $$j_{n}=\frac{j_{v}\cdot p}{R\cdot T}$$

where *j*_*n*_ represents the molar mass flow, *j*_*v*_ [m^3^ s^−1^] is the volumetric flow determined by the volume meter, *p* (95828 Pa) is the pressure, *T* (293.17 K) is the temperature, and *R* (8.314 m^3^ Pa K^−1^ mol^−1^) is the gas constant.

Liquid products dissolved in the catholyte were analyzed after each experiment using proton nuclear magnetic resonance spectroscopy (^1^H‐NMR) with water suppression (Bruker Avance III HD 500 MHz NMR spectrometer). Sodium trimethylsilylpropanesulfonate (DSS) was used as the internal standard for ethanol and propanol quantification, while potassium hydrogen phthalate (KHP) served as the internal standard for formic acid quantification. The average FE for liquid products was determined over the entire experiment duration using the following equation:

(3) $$FE_{i}=\frac{z_{i}*n_{i}*F}{I*t}$$

where *FE*_*i*_, *z*_*i*_, *I*, and *F* are defined as above, *t* [s] is the total experiment time, and *n*_*i*_ [mol] is the amount of substance determined by NMR.

To determine the excess of CO_2_ relative to the total amount of C_2_H_4_ that could be formed at the catalyst interface ($\lambda_{C_{2}H_{4}}$), the amount of accessible CO_2_ in the absorbent solution was compared to the theoretical amount of CO_2_ needed to produce C_2_H_4_ at an FE of 100%. The following equation was used for this calculation:

(4) $$\lambda_{C_{2}H_{4}}=\frac{z_{C_{2}H_{4}}*F*j_{v}\cdot c}{I}$$

Here $\lambda_{C_{2}H_{4}}$ is the resulting lambda factor, $z_{C_{2}H_{4}}$ are the 12 electrons required for the CO_2_ conversion to C_2_H_4_, *F* is the Faraday constant (96485 C mol^−1^), and *I* [A] is the current. *j*_*v*_ [L s^−1^] is the catholyte flow rate supplied to the cell via micro gear pumps, and c is the concentration of the accessible CO_2_ within the supplied absorbent solution (21 mmol L^−1^ [14]).

## Supporting Information

Additional supporting information can be found online in the Supporting Information section. **Supporting** **Fig.** **S1:** REM image of a GDE cross‐section, showing the catalyst layer at the top on the microporous layer, with the macropores layer visible at the bottom. **Supporting Fig. S** **2:** Picture of the cathodic flow field of the eChemicles cell, used in the standard electrolysis setup. **Supporting Fig. S** **3:** Variation of the current density during integrated CO_2_ electrolysis in the range of 50 to 200 mA cm^−2^. Each bar represents the median of the FE of three independent experiments after 3.5 h of electrolysis with the range indicated by the error bars. The electrolysis was performed at 1 bar(a) (absolute pressure), 25°C, a catholyte flow rate of 0.4 g s^−1^, and in the standard electrolyzer setup. It can be observed that as the current density increases, the yield of carbon‐containing products‐particularly C_2_H_4_ decreases, while the HER becomes more prominent. Additionally, the total FE declines with higher current densities. This is most likely due to increased gas evolution, which may promote gas leakage (e.g., through the membrane), resulting in some products not being detected at the regular catholyte outlet.

## Author Contributions

**Fabian Hauf**: conceptualization (lead); investigation (lead); methodology (lead); visualization (lead); writing – original draft (lead). **Ricarda Kollmuß**: supervision (lead); writing – review and editing (lead). **Stefan Haufe**: project administration (equal); writing – review and editing (supporting). **Elias Klemm**: project administration (equal); supervision (supporting); writing – review and editing (supporting).

## Funding

This study was supported by the German Federal Ministry of Research, Technology, and Space under the funding code (03SF0705H).

## Conflicts of Interest

The authors declare no conflicts of interest.

## Supporting information

Supplementary Material

## Data Availability

The data that support the findings of this study are available from the corresponding author upon reasonable request.
