# Supplementary material for: Evaluation of Process Parameters for Integrated CO2 Electrolysis to Produce Ethylene
Source: ChemistryOpen. 2026 Feb 1;15(2):e202500611. doi: 10.1002/open.202500611 (PMC12862004; doi:10.1002/open.202500611)
Supplement: Supplementary file 1 — Supplementary Material [file OPEN-15-e202500611-s001.pdf]

ChemistryOpen

Supporting Information

Evaluation of Process Parameters for Integrated CO_2 Electrolysis to Produce Ethylene

Fabian Hauf, Ricarda Kollmuß, Stefan Haufe and Elias Klemm

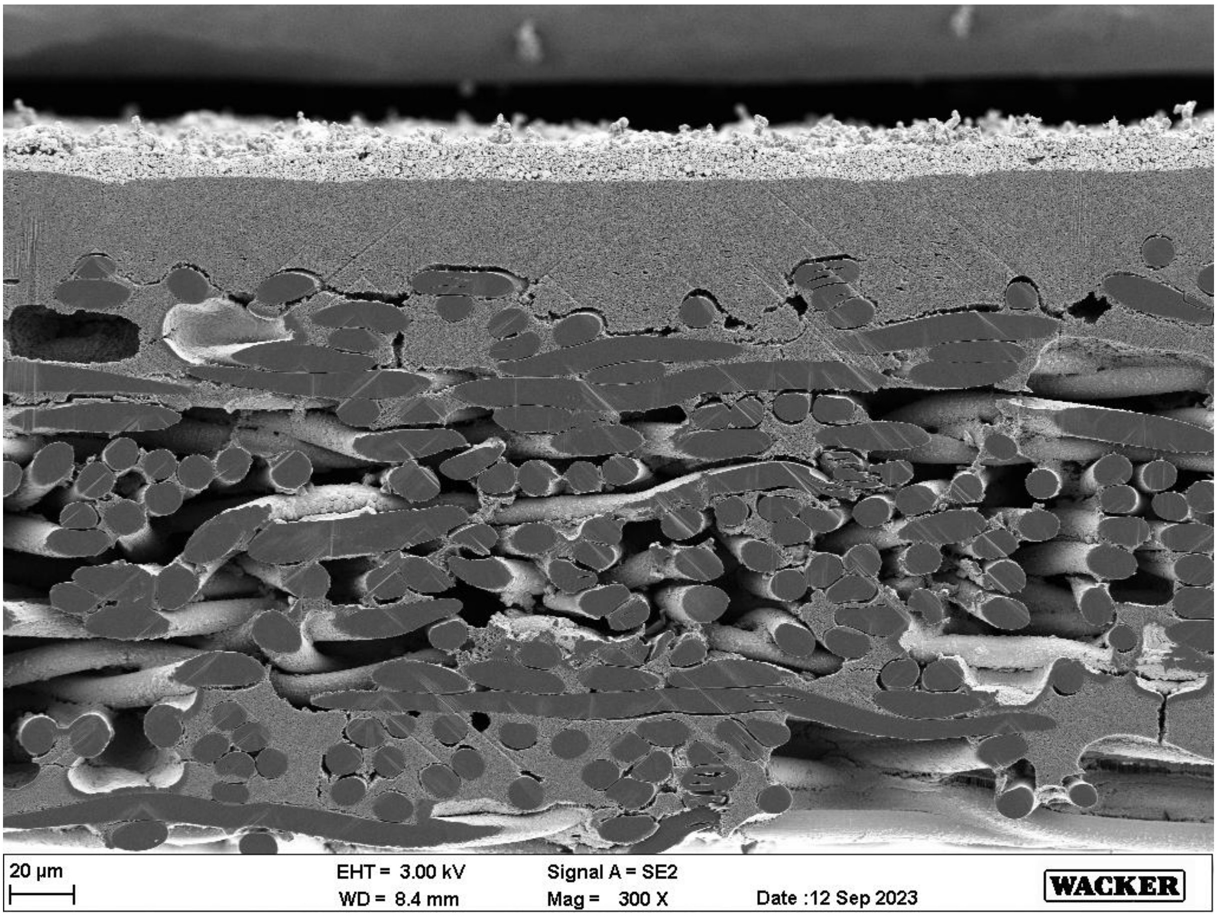

Figure S1: REM image of a GDE cross-section, showing the catalyst layer at the top on the microporous layer, with the macropores layer visible at the bottom.

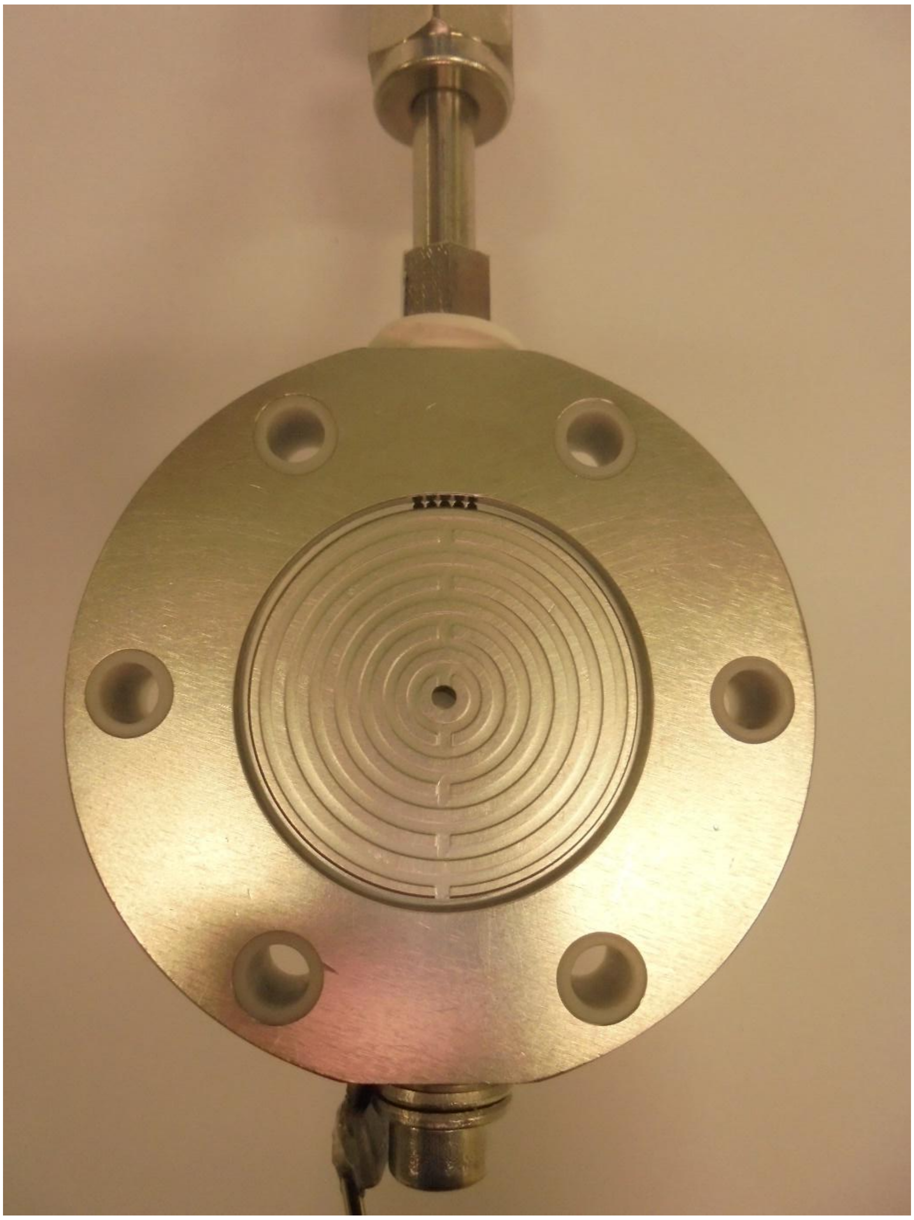

Figure S2: Picture of the cathodic flow field of the eChemicles cell, used in the standard electrolysis setup.

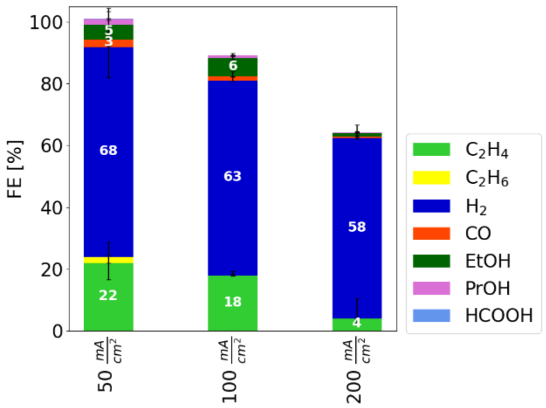

Figure S3: Variation of the current density during integrated CO_2 electrolysis in the range of 50 to 200 mA cm^{-2}. Each bar represents the median of the FE of three independent experiments after 3.5 h of electrolysis with the range indicated by the error bars. The electrolysis was performed at 1 bar(a) (absolute pressure), 25 °C, a catholyte flow rate of 0.4 g s^{-1}, and in the standard electrolyzer setup. It can be observed that as the current density increases, the yield of carbon-containing products-particularly C_2H_4—decreases, while the HER becomes more prominent. Additionally, the total FE declines with higher current densities. This is most likely due to increased gas evolution, which may promote gas leakage (e.g., through the membrane), resulting in some products not being detected at the regular catholyte outlet.
